# Supplementary figures and images for: Perturbations in dynamical models of whole-brain activity dissociate between the level and stability of consciousness
Source: PLoS Comput Biol. 2021 Jul 27;17(7):e1009139. doi: 10.1371/journal.pcbi.1009139 (PMC8315553; doi:10.1371/journal.pcbi.1009139)

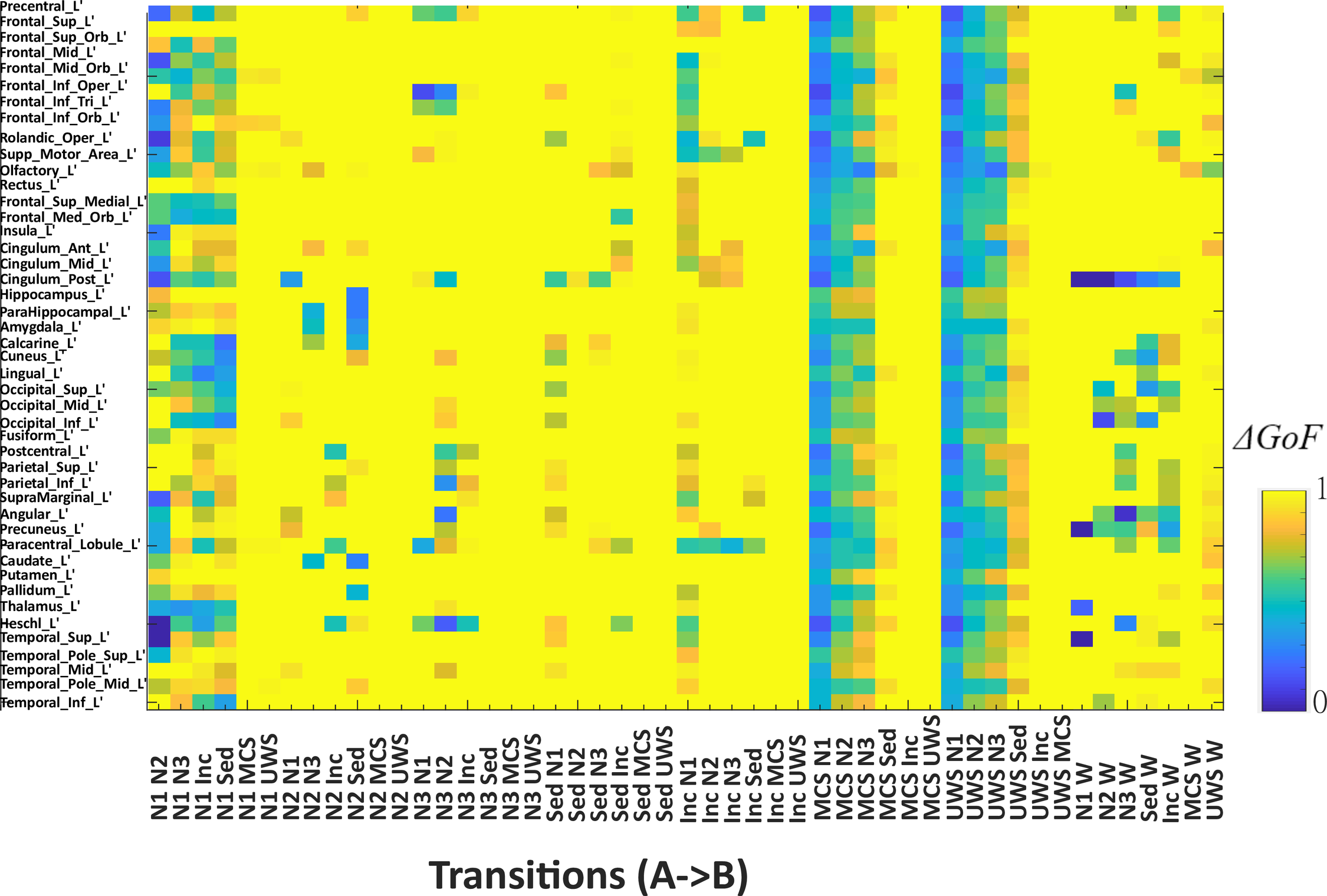

Supplement: S1 Fig — This likelihood is obtained using the ΔGoF, which measures the similarity between the functional connectivity (FC) of the target and the perturbed brain state. Low values of ΔGoF represent similar FC between target and perturbed state, and thus a high likelihood of inducing a transition between both states. The x-axis lists all the possible transitions between the assessed states of consciousness, the y-axis contains the name of the regions in the AAL parcellation, and the color scale indexes ΔGoF. (TIF) [file pcbi.1009139.s003.tif]
